# Subthalamic Stimulation Inhibits Bladder Contraction by Modulating the Local Field Potential and Catecholamine Level of the Medial Prefrontal Cortex

**DOI:** 10.3389/fnins.2020.00917

**Published:** 2020-09-03

**Authors:** Tatsuya Yamamoto, Ryuji Sakakibara, Tomoyuki Uchiyama, Satoshi Kuwabara

**Affiliations:** ^1^Department of Rehabilitation Sciences, Chiba Prefectural University of Health Sciences, Chiba, Japan; ^2^Department of Neurology, Chiba University Graduate School of Medicine, Chiba, Japan; ^3^Division of Neurology, Department of Internal Medicine, Sakura Medical Center, Toho University, Sakura, Japan; ^4^Department of Neurology, International University of Health and Welfare, Ichikawa, Japan

**Keywords:** Parkinson’s disease, subthalamic nucleus deep brain stimulation, local field potential, catecholamine, medial prefrontal cortex, bladder contraction

## Abstract

**Aims:**

The patients with Parkinson’s disease (PD) present with lower urinary tract symptoms (LUTS), but the efficacy of subthalamic nucleus deep brain stimulation (STN-DBS) on LUTS is unknown. The medial prefrontal cortex (mPFC) is a known higher micturition center which are modulated by STN-DBS. We aim to clarify STN-DBS-related changes in the neuronal activity of the mPFC in terms of bladder contraction, using normal and PD rats.

**Methods:**

Experiments in normal and 6-hydroxydopamine hemi-lesioned PD rats were conducted under urethane anesthesia. STN-DBS was applied to the left STN, with simultaneous monitoring of bladder contractions. The mPFC’s local field potential (LFP) was recorded before, during, and after STN-DBS (*n* = 6: normal rats, *n* = 6: PD rats). Before, during and after STN-DBS (*n* = 5: normal rats, *n* = 6: PD rats), extracellular fluid was collected from mPFC.

**Results:**

STN-DBS significantly increased bladder inter-contraction interval. STN-DBS significantly decreased mPFC alpha power in normal rat and increased alpha power in PD rat. The mPFC levels of levodopa, dopamine, serotonin and their metabolites in PD rats decreased significantly during and after STN-DBS, whereas the serotonin and its metabolites and homovanillic acid (HVA) levels decreased significantly in normal rats following STN-DBS.

**Conclusion:**

STN-DBS increased intercontraction intervals for the bladder in both normal and PD rats, as assessed by alpha power and catecholamine levels in mPFC, probably due to changes in neural activity. The effect of STN-DBS on mPFC levels of catecholamine differentiated between the normal and PD rats.

## Introduction

Subthalamic nucleus deep brain stimulation (STN-DBS) is known to dramatically alleviate motor complications of advanced-stage Parkinson’s disease (PD) (Okun, 2012). However, despite the dramatic improvement in motor dysfunctions, the quality of life (QOL) is not significantly improved by STN-DBS (Yamamoto et al., 2017). One possible explanation for this discrepancy is that the patients’ QOL is also influenced by non-motor symptoms (Fabbri et al., 2017). Although, it has been well known that STN-DBS does improve non-motor symptoms (Dafsari et al., 2016), some non-motor symptoms deteriorated and might contribute to worse QOL after STN-DBS. It is well known that several non-motor symptoms are prevalent and severe among patients with advanced-stage PD (Kalia and Lang, 2015), including lower urinary tract symptoms (LUTS), which may contribute significantly to the QOL deterioration (Sakakibara et al., 2016).

Although the pathophysiological mechanisms underlying PD-related LUTS are not fully understood, it is possible that they involve dysfunctions of higher micturition centers such as the medial prefrontal cortex (mPFC) and the basal ganglia (Sakakibara et al., 2012). We have previously reported that, in cats, some nuclei in the basal ganglia such as the striatum (Yamamoto et al., 2009), substantia nigra (Sakakibara et al., 2002), and STN (Sakakibara et al., 2003) are involved in bladder contraction, and most recorded neurons fire preferentially during the isovolumetric bladder relaxation phase, suggesting that they inhibit bladder contraction (Sakakibara et al., 2002, 2003; Yamamoto et al., 2009). In fact, electrical stimulation of these neurons inhibits bladder contraction (Herzog et al., 2006, 2008; Horn et al., 2017). We have also reported that most recorded mPFC neurons fire preferentially during the isovolumetric bladder relaxation phase in normal cats (Yamamoto et al., 2010).

Recent functional imaging studies showed the mPFC is involved in the voluntary control of the micturition reflex and is thought to strongly inhibit it via periaqueductal gray (PAG) during the storage phase (Fowler et al., 2008; Griffiths, 2015). Therefore, mPFC dysfunctions may lead to LUTS especially overactive bladder which are prevalent in PD.

Several reports revealed that STN-DBS increased bladder capacity or inhibited detrusor overactivity in some PD patients leading to improvement in LUTS (Seif et al., 2004; Winge et al., 2007; Mock et al., 2016; Witte et al., 2018). Herzog et al. demonstrated that STN-DBS in PD patients normalizes the activity of the anterior cingulate cortex and prefrontal cortex, which are usually overactive in those patients, thereby improving LUTS. Herzog et al. (2006, 2008) also reported that STN-DBS might normalize the afferent urinary information and thereby leading to the improvement of overactive bladder in PD patients.

However, the exact mechanisms by which STN-DBS ameliorate PD-related LUTS remains unknown.

Because, STN do not directly project to PAG regulating micturition reflex, it is unlikely that STN-DBS directly affect micturition reflex via PAG.

Since the output signals of the basal ganglia project into the mPFC via thalamus and given that STN-DBS significantly influences the activity of the output signals of the basal ganglia which project to thalamus and ultimately to mPFC, STN-DBS may influence the activity of mPFC thereby changing bladder inter-contraction interval.

Because mPFC have dense projections to PAG which regulate micturition reflex (Fowler et al., 2008; Griffiths, 2015), it is reasonable to hypothesize that STN-DBS influence the activity of mPFC, which might result in changing bladder inter-contraction interval.

The present study aims to clarify the changes in the mPFC neuronal activity induced by STN-DBS and their relation to bladder contraction/relaxation cycle in a rat model of PD. For that purpose, we recorded the mPFC local field potential (LFP) and measured mPFC catecholamine levels. The LFP is obtained by extracellular recordings with low pass filtering. LFP represents the mean field potential generated in the vicinity of the recording electrode by the slow components of synaptic potentials. The power from LFP was usually measured through spectral analysis, which is widely used in clinical neurology. For example, increased beta power in STN or motor cortex are known to be negatively correlated with motor functions (Horn et al., 2017). STN-DBS is known to substantially reduce the beta-power in patients with STN or cerebral cortex, thereby improving motor symptoms (Brittain et al., 2014). The levels of catecholamine were measured since the mPFC receives dense catecholaminergic projections from the ventral tegmental area (Sakakibara et al., 2002) and raphe nuclei (Ito et al., 2006), which produce dopamine and serotonin, known to regulate the micturition reflex (Sakakibara et al., 2002; Ito et al., 2006).

## Materials and Methods

### Animals and Ethic Statement

All experiments were carried out on adult female Sprague – Dawley (SD) rats (14–16 weeks old, weighing 200–300 g), in accordance with the Laboratory Animals Care and Use Guideline. All efforts were made to minimize the suffering of the animals and reduce the number of animals used. The experimental protocol was approved by the Animal Ethics Committee, Chiba University Graduate School of Medicine (April 1st 2019, number 1-349). The animals were housed in a room with an alternating 12-h light/dark cycle under standard environmental conditions.

### 6-Hydroxydopamine (6-OHDA) Lesion (PD Model)

Sprague – Dawley rats were operated under a sodium pentobarbital anesthesia (40 mg/kg, intraperitoneally). The animals received a 2 μg/ml 6-OHDA unilateral injection (Sigma – Aldrich, Japan) dissolved in 5 μl of 0.9% sterile saline containing 0.1% ascorbic acid in the left medial forebrain bundle at a rate of 1 μl/min. In relation to bregma, the stereotaxic coordinates of the injection site were as follows: anteroposterior, −3.6 mm; lateral, 2.0 mm; and dorsoventral, −8.8 mm.

### Motor Behavior

The extent of DA-neuron lesion was evaluated 2 weeks after 6-OHDA injection, through apomorphine challenge (1 mg/kg, intraperitoneally; Sigma–Aldrich). In animals that performed > 80 net contraversive rotations in 20 min the lesion was considered successful.

### Recordings of Isovolumetric Bladder Contraction

Recordings of isovolumetric bladder contraction were performed under urethane anesthesia (0.7 g/kg, intraperitoneally). The depth of anesthesia was monitored by the absence of response to toe pinch. If the responses to toe pinch was found, one-third of the initial dose of urethane was injected. *Trans*-urethrally, a single lumen catheter was inserted into the bladder to measure the bladder pressure. The catheter was attached to a syringe pump with an in-line pressure transducer. Saline was instilled (100 μL/min) to maintain isovolumetric spontaneous bladder contraction and bladder pressure was recorded (Urolab, Lifetech, United States). Bladder inter-contraction intervals were measured before (30 min), during (30 min), and after (30 min) STN-DBS.

### STN-DBS and Extracellular mPFC Recordings

STN-DBS and extracellular mPFC recordings were conducted under urethane anesthesia in normal (*n* = 6) and PD (*n* = 6) rats (0.7 g/kg, intraperitoneally). The depth of anesthesia was monitored by the absence of response to toe pinch. If the responses to toe pinch was found, one-third of the initial dose of urethane was injected. In PD rats, the experiments were carried out 30–40 days after 6-OHDA injection.

A concentric bipolar platinum/iridium stimulating electrode (outer diameter: 125 μm; Pt/Ir; FHC, United States) was inserted stereotaxically into left STN. In relation to bregma, the stereotaxic coordinates were as follows: anteroposterior, −3.8 mm; lateral, 2.4 mm; and dorsoventral, −8.1 mm.

The parameters for the stimulation were: frequency, 130 Hz; intensity, 200 μA; pulse width, 80 μs; and stimulation time, 30 min. Electrical rectangular biphasic stimulation was applied with a STG-4004 stimulator (Multi Channel Systems, Germany).

Extracellular mPFC LFP recordings were performed with the same Pt/Ir electrode (outer diameter, 125 μm; tip impedance, 9–12 MOhm) before (30 min), during (30 min) and after (30 min) stimulation. The stereotactic coordinates were as follows in relation to bregma: anteroposterior + 2.2 mm; lateral 0.8 mm; and dorsoventral −4.0 mm. Extracellular recordings between each pole of the concentric bipolar electrode were performed and extracellular signals were recorded (band-pass filtered, 0.3 Hz–10 kHz) and amplified (×10,000) through a high-performance extracellular amplifier (DAGAN 2400A, DAGAN, United States). In the STN and mPFC an electrical lesion was created at the end of each experiment.

### Power Spectrum Analysis

Off-line analysis of the mPFC power spectrum was done using the Lab Chart software (AD Instrument, Australia). Fast Fourier transforms (FFTs) were performed to analyze STN LFP on a 0.3–50 Hz frequency domain. Power spectral densities (PSDs) with 131072 FFT size, Hann window, and a 50 percent overlap were estimated, and log10 (PSD) normalized.

### *In vivo* Microdialysis and High-Performance Liquid Chromatography (HPLC)

In SD (*n* = 5) and PD (*n* = 6) rats, extracellular fluid was collected from the mPFC before, during, and after subthalamic stimulation. A concentric I-type dialysis probe (diameter, 0.22 mm; exposed membrane, 2.0 mm; A-I-12-02; Eicom Inc., Kyoto, Japan) was stereotaxically inserted into the mPFC, ipsilateral to the site of STN stimulus. In relation to bregma, the stereotaxic coordinates were as follows: anteroposterior, +2.2 mm; lateral 0.8 mm; and dorsoventral −4.0 mm. The perfusion rate was maintained at 2 μl/min using modified Ringer’s solution (Na^+^, 147 mM; K^+^, 4 mM; Ca^2+^, 2.3 mM; and Cl^–^, 155.6 mM). Dialysates were collected 1 h after implantation of the dialysis probe. The collection was carried out before (30 min), during (30 min), and after (30 min) subthalamic stimulation with high frequencies. The dialysates were collected at 10-min intervals for 1.5 h and stored at −80°C. The average levels of catecholamines in the dialysates collected during the first 10, 20, and 30 min prior to stimulation were defined as basal, and the levels at the following points were evaluated as the ratios to the basal levels conformed to our previous study (Yamamoto et al., 2014). The HPLC system used for catecholamine determination was equipped with an electrochemical detector system (HTEC500; Eicom), and the mobile phase used was 0.1 M citric acid–0.1 M sodium acetate (pH 3.9) containing 140 mg/L sodium 1-octane sulfonate, 5 mg/L EDTA-2Na, and 15% methanol at a flow rate of 0.23 ml/min. The samples were injected manually into an analytics column (EICOMPAK SC-5ODS; 2.1 ϕmm × 150 mm; Eicom). Electrochemically, catecholamine level was detected using a graphite electrode (WE-3G; Eicom) at 700 mV relative to a silver/silver chloride reference electrode.

### Histopathological Examination

Brain tissues were fixed, embedded in paraffin, in a 10 percent neutral formalin solution and cut into 10-μm sections using conventional techniques. The sections were stained with cresyl violet to confirm the electrode location in the STN, and the mPFC probe.

### Immunohistochemistry for Tyrosine Hydroxylase

Immunohistochemical staining of the brain sections was performed using primary anti-tyrosine hydroxylase antibody developed in rabbits (T8700; Sigma – Aldrich), using the avidin – biotin complex method. After xylene deparaffinization and gradual dehydration, 0.5 per cent hydrogen peroxide (H_2_O_2_) blocked endogenous peroxidase activity for 15 min. In phosphate-buffered saline (PBS) with diluted primary antibody (1:1000), tissue sections were then incubated with 10 percent normal goat serum (G9023; Sigma – Aldrich) overnight at 4° C. They were then washed as a secondary antibody in PBS containing 0.05 percent Tween-20 (PBST), incubated overnight at 4° C with biotinylated anti-rabbit immunoglobulin G antibody raised in goats (BA-1000; Vector Labs; 1:1000), and washed again in PBST. The sections were washed in PBST after incubation with the Vectastain ABC Reagents (PK-6100; Vector Labs; 1:1000) for 2 h, and then visualized for 10 min by reaction with 3,3-diaminobenzidine tetrahydrochloride (Sigma – Aldrich) and 0.03 percent H_2_O_2_ in Tris-buffered saline.

### Statistical Analysis

All data were expressed as means ± standard error of mean (SEM). The statistical analysis was done using the SPSS version 22.0 software (IBM, Armonk, NY, United States). Dunnett’s tests were used to compare mPFC levels of catecholamine with those before stimulation (basal levels) during and after STN stimulation, to determine the effects of STN-DBS. *P*-values < 0.05 have been deemed statistically significant. To analyze the effect of STN-DBS on the mean logarithmic power in alpha frequency (8–13 Hz), Dunnett’s tests were used to compare the mean logarithmic power in alpha frequency “during” and “after” STN-DBS with those “pre” STN-DBS in normal and PD rats. The paired *t*-test was performed for the comparison of the mean logarithmic power in alpha frequency between bladder contraction phase and relaxation phase.

To analyze the effect of STN-DBS on the bladder inter-contraction interval, the Mann–Whitney U test was performed between normal and PD rats “before,” “during,” and “after” STN-DBS.

## Results

### Histological Confirmation of the STN Electrode and 6-OHDA Lesion to the Substantia Nigra

**FIGURE 1:**
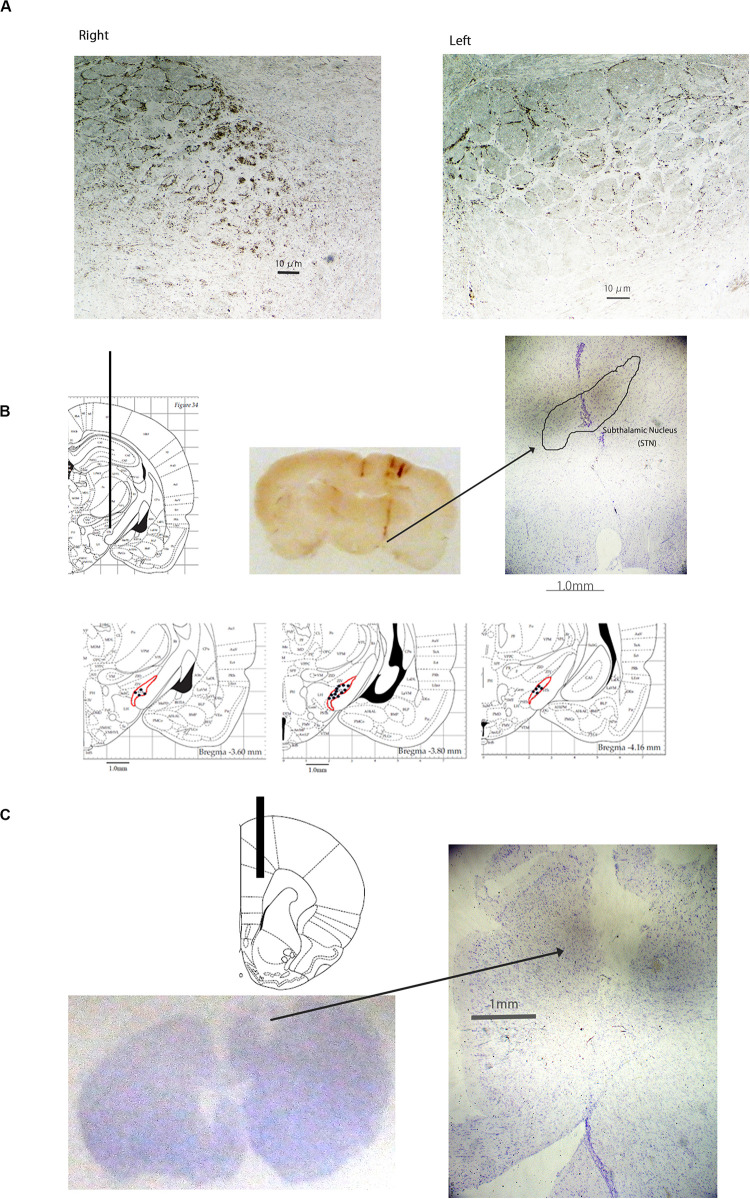
Photographs of tyrosine hydroxylase-immunostained coronal rat brain sections of the substantia nigra **(A)**, Cresyl Violet-stained coronal rat brain sections of the subthalamic nucleus (STN) **(B)**, and Cresyl Violet-stained coronal section of the medial prefrontal cortex (mPFC). The number of dopaminergic cells was significantly decreased in the left substantia nigra **(A)**. The location of the electrode in the STN **(B)** is presented. The location of the probe in the mPFC **(C)** is presented.

Photographs of the coronal rat sections of tyrosine hydroxylase (TH)-immunostained brain showed significant reduction of dopaminergic cells in the left substantia nigra (Figure 1A). The total number of TH positive cells in lesioned side was 1490 ± 414.4, whereas in unaffected side was 3999 ± 920.8. The locations of the STN electrode (Figure 1B) and the mPFC probe (Figure 1C) were determined by histological staining with cresyl violet.

### Effects of STN-DBS on the Bladder Inter-Contraction Intervals

**FIGURE 2:**
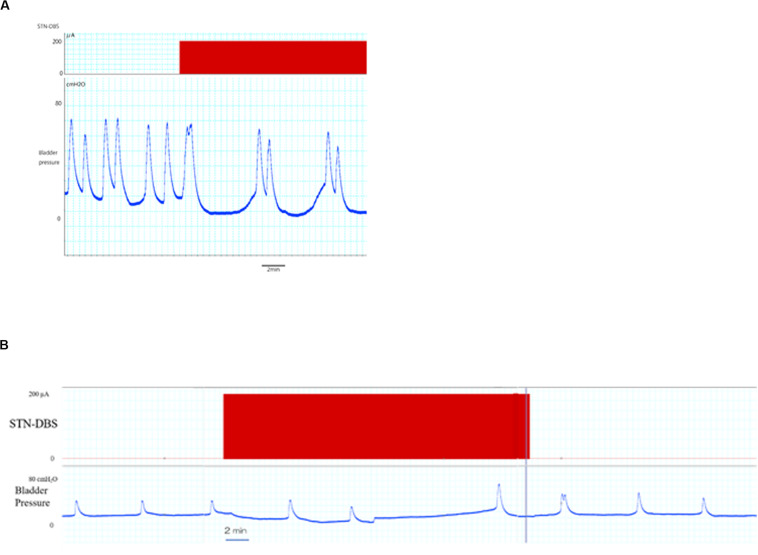
Effects of STN-DBS on bladder inter-contractions interval. **(A)** The upper and lower traces represent the STN-DBS (subthalamic nucleus deep brain stimulation) and bladder pressure, respectively. STN-DBS increased the bladder inter-contractions interval in PD rat. **(B)** An entire trace of bladder pressure including 30 min stimulation are represented. The elongated bladder inter-contractions interval during stimulation tended to decrease after stimulation in PD rat.

The typical bladder contraction responses induced by STN-DBS are represented in Figure 2. STN-DBS significantly increased the inter-contraction intervals in normal rats from 155,46 ± 23,45 s (pre-STN-DBS) to 260,43 ± 39,18 s (during STN-DBS) (*p* < 0,01; Table 1), and from 175.28 ± 16.39 s (pre STN-DBS phase) to 231.18 ± 26.32 s (during STN-DBS phase) in PD rats (*p* < 0.05; Table 1). Although, no significant differences were found between normal and PD rats regarding the bladder inter-contraction intervals “before” and “after” STN-DBS, bladder inter-contraction interval “during” STN-DBS was significantly shorter in PD rats than that in normal rats.

**TABLE 1:** Bladder inter-contraction interval.

	**Pre-STN-DBS (30 min)**	**During STN-DBS (30 min)**	**Post-STN-DBS (30 min)**	***p*-value (pre vs. during)**	***p*-value (during vs. post)**
PD rat	175.28 ± 16.39 (10.26 ± 0.96 cycles)	231.18 ± 26.32 (7.78 ± 0.89 cycles)	221.43 ± 25.06 (8.12 ± 0.93 cycles)	*p* = 0.05	*p* = 0.06
Normal rat	155.46 ± 23.45 (11.57 ± 1.78 cycles)	260.43 ± 39.18 (6.91 ± 1.06 cycles)	209.47 ± 36.31 (8.59 ± 1.53 cycles)	*p* = 0.01	*p* = 0.09
*p*-value (Normal vs. PD)	*p* = 0.17	*p* = 0.03	*p* = 0.17		

*Results presented in seconds (s) and as Mean ± SEM.*

Increased bladder inter-contraction interval induced by STN-DBS tended to decrease after DBS in PD and normal rats (Table 1).

### Effects of STN-DBS on mPFC Spectrum With Relation to Bladder Contraction/Relaxation Phase

**FIGURE 3:**
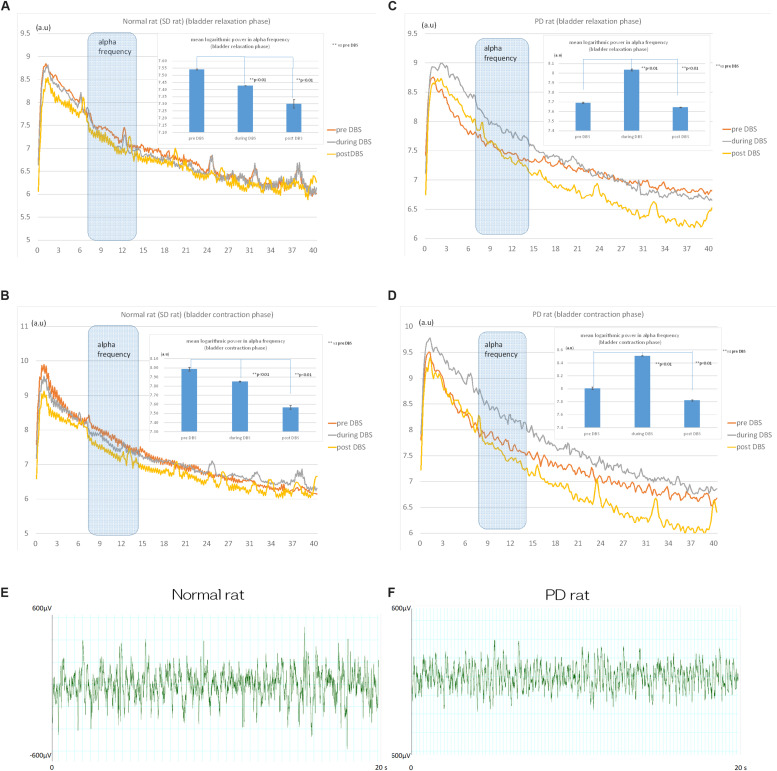
Effects of STN-DBS on the spectrum of mPFC in normal (SD) and PD rat. **(A)** Power spectrum analysis revealed that STN-DBS significantly decreased the mean logarithmic power in mPFC alpha frequency (8–15 Hz) from 7.54 ± 0.05 (a.u.) to 7.42 ± 0.01 (a.u.) during DBS (*p* < 0.01) and to 7.30 ± 0.03 (a.u.) post DBS in Normal rats (*p* < 0.01) during bladder relaxation phase. **(B)** STN-DBS significantly decreased the mean logarithmic power in mPFC alpha frequency (8–15 Hz) from 7.98 ± 0.02 (a.u.) to 7.85 ± 0.06 (a.u.) during DBS (*p* < 0.01) and to 7.56 ± 0.02 (a.u.) post DBS in Normal rats (*p* < 0.01) during bladder contraction phase. **(C)** STN-DBS significantly increased the mean logarithmic power in mPFC alpha frequency (8–15 Hz) from 7.69 ± 0.08 (a.u.) to 8.03 ± 0.01 (a.u.) during DBS (*p* < 0.01) and decreased to 7.64 ± 0.04 (a.u.) post DBS in PD rats (*p* < 0.01) during bladder relaxation phase. **(D)** STN-DBS significantly increased the mean logarithmic power in mPFC alpha frequency (8–15 Hz) from 8.00 ± 0.02 (a.u.) to 8.50 ± 0.01 (a.u.) during DBS (*p* < 0.01) and decreased to 7.82 ± 0.01 (a.u.) post DBS in PD rats (*p* < 0.01) during bladder contraction phase. The raw recording of the mPFC activity in normal and PD were in **(E)** and **(F)**, respectively.

Analysis of the power spectrum showed that STN-DBS decreased the mean logarithmic power in the mPFC alpha frequency (8–15 Hz) significantly from 7.54 ± 0.05 (a.u.) to 7.42 ± 0.01 (a.u.) during DBS (*p* < 0.01) and to 7.30 ± 0.03 (a.u.) post DBS in normal rats (*p* < 0.01; Figure 3A) during bladder relaxation phase. In addition, the mean logarithmic power at mPFC alpha frequency (8–15 Hz) was significantly reduced by STN-DBS from 7.98 ± 0.02 (a.u.) to 7.85 ± 0.06 (a.u.) during DBS (*p* < 0.01) and to 7.56 ± 0.02 (a.u.) post DBS in normal rats (*p* < 0.01; Figure 3B) during bladder contraction phase.

STN-DBS increased the mean logarithmic power at mPFC alpha frequency (8–15 Hz) significantly from 7.69 ± 0.08 (a.u.) to 8.03 ± 0.01 (a.u.) during DBS (*p* < 0.01); and decreased to 7.64 ± 0.04 (a.u.) post DBS in PD rats (*p* < 0.01; Figure 3C) during bladder relaxation phase. STN-DBS increased the mean logarithmic power at the mPFC alpha frequency (8–15 Hz) significantly from 8.00 ± 0.02 (a.u.) to 8.50 ± 0.01 (a.u.) during DBS (*p* < 0.01) and decreased to 7.82 ± 0.01 (a.u.) post DBS in PD rats (*p* < 0.01; Figure 3D) during bladder contraction phase.

The raw recording of the mPFC activity in normal and PD were in Figures 3E,F, respectively.

### Comparison of the Mean Logarithmic Power of the Alpha Frequency Between Bladder Contraction and Relaxation Phase

The mean logarithmic power of alpha frequency in bladder contraction phase was significantly larger than that in bladder relaxation phase in SD and PD rat.

[SD rat: pre DBS: relaxation phase 7.54 ± 0.05 (a.u.), contraction phase 7.98 ± 0.02 (a.u.) *p* < 0.01, during DBS: relaxation phase 7.42 ± 0.01 (a.u.), contraction phase 7.85 ± 0.06 (a.u.) p < 0.01, post DBS: relaxation phase 7.30 ± 0.03 (a.u.), contraction phase 7.56 ± 0.02 (a.u.) *p* < 0.01.

PD rat: pre DBS: relaxation phase 7.69 ± 0.08 (a.u.), contraction phase 8.00 ± 0.02 (a.u.) *p* < 0.01, during DBS: relaxation phase 8.03 ± 0.01 (a.u.), contraction phase 8.50 ± 0.10 (a.u.) *p* < 0.01, post DBS: relaxation phase 7.64 ± 0.04 (a.u.), contraction phase 7.82 ± 0.01 (a.u.) *p* < 0.01].

### Comparison of the Mean Logarithmic Power of the Alpha Frequency Between SD Rat and PD Rat

The mean logarithmic power of alpha frequency in PD rat was significantly larger than SD rat during bladder contraction and relaxation phase except for during bladder contraction phase before STN-DBS.

[pre DBS: relaxation phase: SD rat 7.54 ± 0.05 (a.u.), PD rat 7.69 ± 0.08 (a.u.) *p* < 0.01, contraction phase: SD rat 7.98 ± 0.02 (a.u.), PD rat 8.00 ± 0.02 (a.u.) *p* = 0.74.

During DBS: relaxation phase: SD rat 7.42 ± 0.01 (a.u.), PD rat 8.03 ± 0.01 (a.u.) *p* < 0.01, contraction phase: SD rat 7.85 ± 0.06 (a.u.), PD rat 8.50 ± 0.10 (a.u.) *p* < 0.01.

Post DBS: relaxation phase: SD rat 7.30 ± 0.03 (a.u.), PD rat 7.64 ± 0.04 (a.u.) *p* < 0.01, contraction phase: SD rat 7.56 ± 0.02 (a.u.), PD rat 7.82 ± 0.01 (a.u.) *p* < 0.01].

### Effects of STN-DBS on Catecholamine Levels Within mPFC

The basal levels of catecholamine (before subthalamic stimulation) in the mPFC are shown in Table 2. Basal levels of LDOPA (L-3,4-dihydroxyphenylalanine), DOPAC (3,4-dihydroxyphenylacetic acid), and DA (dopamine) were significantly lower in PD than in normal rats, while basal levels of HVA (homovanillic acid), 5HIAA (5-hydroxyindole acetic acid), and 5HT (5-hydroxytryptamine) were found to be no different.

**TABLE 2:** Basal catecholamine levels.

**(pg/μl)**	**PD rat (*n* = 6)**	**Normal rat (*n* = 5)**	***P*-value (PD vs. SD)**
LDOPA	3.61 ± 0.05	137.18 ± 83.2	*p* = 0.047
DOPAC	2.00 ± 0.53	21.63 ± 7.7	*p* = 0.003
DA	1.00 ± 0.35	13.36 ± 3.80	*p* = 0.01
5HIAA	1.16 ± 0.42	2.90 ± 0.51	*p* = 0.016
HVA	26.05 ± 4.05	16.36 ± 4.51	*p* = 0.13
5HT	1.78 ± 0.49	4.22 ± 1.76	*p* = 0.12

*Results presented as Mean ± SEM.*

**FIGURE 4:**
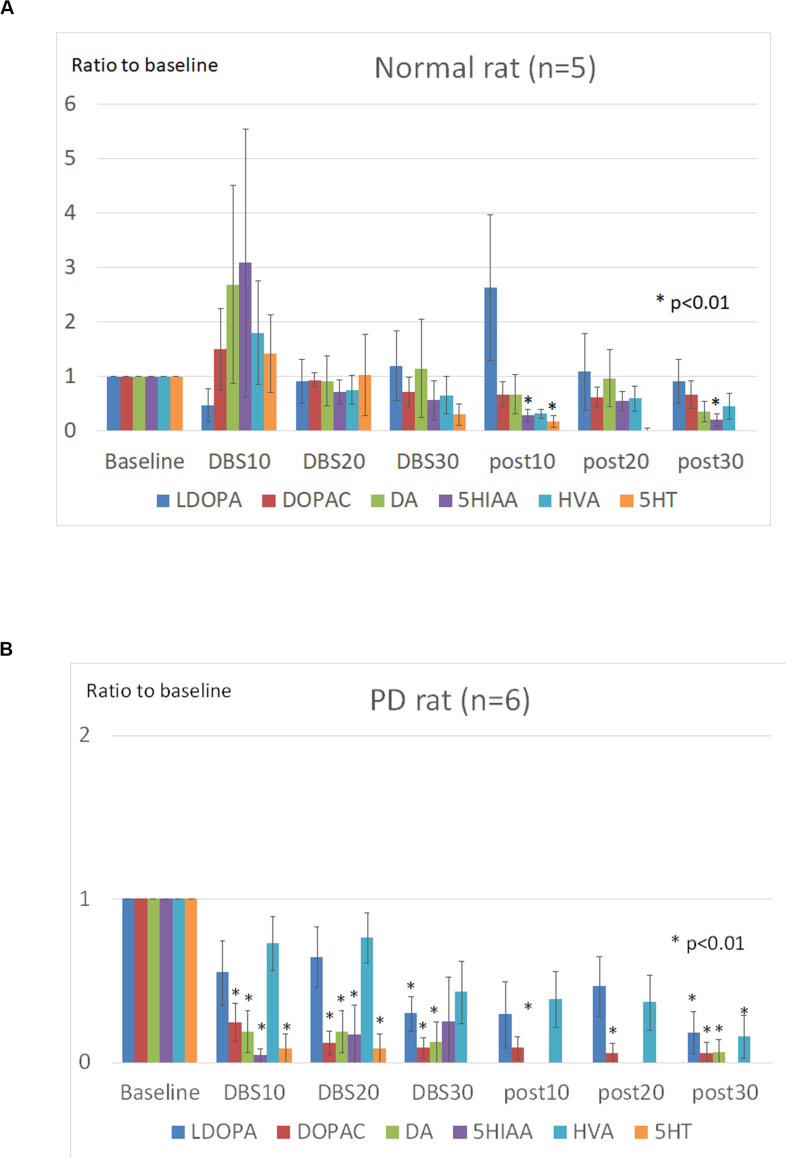
Effects of STN-DBS on the mPFC catecholamines. The levels at the following points were represented as the ratios of the basal levels which was the average levels of catecholamines during the first 30 min before stimulation. **(A)** Normal (SD) rats. The levels of 5-HIAA (5-hydroxyindole acetic acid) and 5-HT (5-hydroxytryptamine) were significantly decreased after STN-DBS (*p* < 0.05), whereas the levels of LDOPA (L-3,4-dihydroxyphenylalanine), DOPAC (3,4-Dihydroxyphenylacetic acid), DA (dopamine), and HVA (homovanillic acid) were not significantly changed during and after STN-DBS. **(B)** PD rats. The levels of LDOPA, DOPAC, and DA were significantly decreased during and after STN-DBS (*p* < 0.01), whereas the levels of HVA were significantly decreased only after STN-DBS (*p* < 0.01). The levels of 5-HIAA and 5-HT were significantly decreased during STN-DBS (*p* < 0.05).

The Dunnet test showed that 5-HIAA and 5-HT levels in normal rats decreased significantly after STN-DBS (*p* < 0.05), while LDOPA, DOPAC, DA and HVA levels remained unchanged during and after STN-DBS (Figure 4A). In PD rats the levels of LDOPA, DOPAC, and DA decreased significantly during and after STN-DBS (*p* < 0.01), while HVA levels decreased significantly only after STN-DBS (*p* < 0.01). During STN-DBS the 5-HIAA and 5-HT levels were significantly reduced (*p* < 0.05) (Figure 4B).

## Discussion

This study shows that STN-DBS significantly increases intercontraction intervals of the bladder in both normal and PD rats, with a significantly higher effect in normal rats indicating that storage dysfunction might be more severe in PD rats. A concomitant STN-DBS-induced change to the mPFC was the significant decrease in the mean logarithmic power of alpha frequency in normal rats and the significant increase in the mean logarithmic power of alpha frequency in PD rat. The mean logarithmic power of alpha frequency in PD rat was significantly larger than SD rat during bladder contraction and relaxation phase except for during bladder contraction phase before STN-DBS indicating that there was a different effect of STN-DBS on mPFC neural oscillation between normal and PD rats. Regarding catecholamine levels, 5-HIAA and 5-HT decreased significantly in normal rats after STN-DBS while dopamine and serotonin and their metabolites decreased significantly in PD rats after STN-DBS. In addition, we found that alpha power in mPFC during bladder contraction phase was significantly larger than that during bladder relaxation phase in normal and PD rat, indicating that mPFC directly regulate bladder contraction/relaxation cycle by changing alpha power.

**FIGURE 5:**
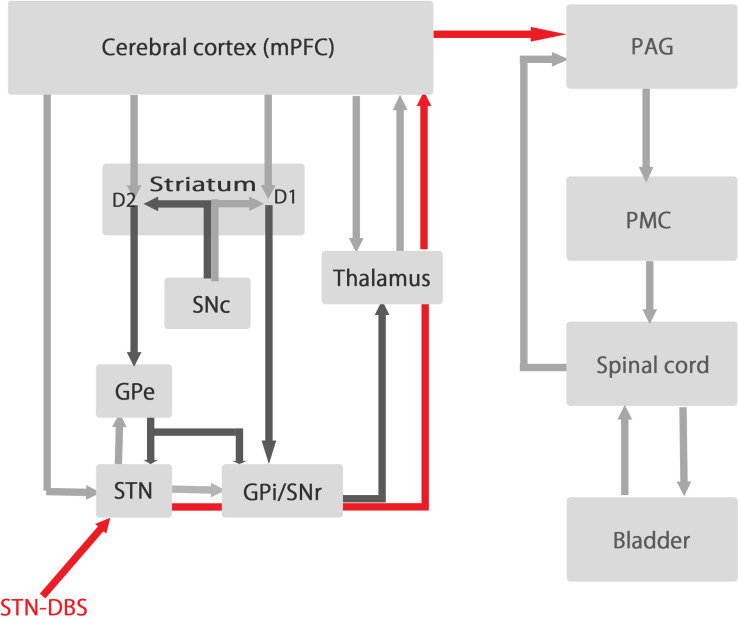
The relationships between the circuit of basal ganglia and the circuit of micturition. The left side represent the circuit of basal ganglia and the right side represent the circuit of micturition. STN-DBS influences the activity of the output signals of the basal ganglia (GPi/SNr) and send signals (red line) to mPFC via thalamus to influence the activity of mPFC which have dense projections to PAG regulating micturition reflex. SNc, substantia nigra pars compacta; SNr, substantia nigra pars reticulate; STN, subthalamic nucleus; GPe, globus pallidum externa; GPi, globus pallidum interna; PAG, periaqueductal gray; PMC, pontine micturition center.

Several previous studies including functional imaging study suggest that the mPFC plays an important role in the regulation of the bladder contraction (Fowler et al., 2008; Griffiths, 2015). We have also reported that most recorded mPFC neurons fire preferentially during the isovolumetric bladder relaxation phase in normal cats (Yamamoto et al., 2010). These evidences suggest that STN-DBS may affect mPFC function significantly through thalamus and antidromical activation of hyper direct pathway, which in turn affect PAG regulating bladder contraction (Figure 5).

The relationship between STN-DBS and mPFC alpha power is important for the interpretation of the present results. It is well known that STN-DBS significantly decreases the beta power of STN and that its degree of reduction is well correlated with the improvement in motor symptoms in PD patients (Brittain et al., 2014). Although there are few reports examining the effect of STN-DBS on mPFC alpha power, some reports demonstrated that mPFC-STN coupling are involved in decision-making, indicating that STN-DBS might modulate mPFC-STN coupling (Delaville et al., 2015). Moreover, higher alpha activity was observed in STN ventromedial region which is known to have strong connections with mPFC (Horn et al., 2017).

PFC is known to play a significant role in determining the socially acceptable times and places to void (Fowler et al., 2008; Griffiths, 2015). Since the STN itself has an important role in regulating bladder contraction (Sakakibara et al., 2003), it is reasonable to hypothesize that STN-DBS modulate mPFC alpha power, thereby influencing mPFC-STN coupling, which in turn leads to the prolongation of bladder inter-contraction intervals during STN-DBS. It is of importance to note that change in alpha power induced by STN-DBS in mPFC was different between normal and PD rat, which might explain the reason why the effect of STN-DBS on the prolongation of bladder inter-contraction interval was significantly low in PD rat. Moreover, recent study reported that beta suppression occurred in the globus pallidus interna (GPi) during urinary voiding but not in the STN (Roy et al., 2018). In PD patients, the beta signal in the GPi during voiding was significantly associated with the extent of incontinence and urinary frequency (Roy et al., 2018). Since output signals of GPi are sent back to mPFC via thalamus, this study indicated that DBS might significantly influence the activity of mPFC in PD patients.

What is important in utilizing alpha power or beta power calculated from spectral analysis of LFP is that LFP is different from spike activity and LFP do not represent the neuronal excitation or inhibition. LFP reflect oscillatory network activities of brain circuit and abnormal oscillatory activities usually correlate with neurological dysfunction such as increased beta power in PD (Okun, 2012).

The effects of the STN-DBS on mPFC catecholamine levels have never been examined in terms of bladder contraction (Stefani et al., 2017). We have previously reported that STN-DBS significantly decreases DOPAC (metabolites of dopamine) in the striatum of PD rats (Yamamoto et al., 2014). Although the effect of STN-DBS on mPFC catecholamine levels is not well elucidated, the present study indicates that STN-DBS decreases both dopamine and serotonin and their metabolites in PD rats, whereas in normal rats only serotonin and its metabolites are decreased. These different responses may indicate that mPFC catecholamines are differentially involved in the regulation of bladder contraction in normal and PD.

There are some limitations in this study. We could not evaluate the direct relationships between the catecholamine levels in mPFC and bladder contraction/relaxation cycle due to low temporal resolution in microdialysis experiment. However, mPFC is well known as a higher micturition center and we showed that mPFC might directly regulate the bladder contraction/relaxation cycle by changing alpha power. Furthermore, it is well known that mPFC receive dense catecholaminergic projections from raphe nucleus (Mock et al., 2016), ventral tegmental area (Sakakibara et al., 2002), which plays a significant role in the regulation of bladder contraction according to our previous studies. Therefore, changes in the levels of catecholamine in mPFC might contribute to the regulation of bladder contraction.

Another limitation is that the direct relationships between the changes in alpha power and changes in neuronal excitations in mPFC are unknown. Because it is well known that mPFC play an important role in regulating bladder contraction and STN have direct projections with mPFC, it is possible to understand that STN-DBS might increase bladder inter-contraction interval probably through modulation of alpha power in mPFC in this study. However, increased or decreased alpha power do not necessarily represent the neuronal excitation or inhibition in mPFC. The relationships between neuronal oscillations and neuronal excitations should be further examined.

Furthermore, we must mention the stimulation parameter (frequency, 130 Hz; intensity, 200 μA; pulse width, 80 μs; and stimulation time, 30 min). The stimulation frequency and pulse width in this study are basically equivalent to clinically used stimulation parameter for PD patients undergoing STN-DBS. It is possible that effect of STN stimulation (intensity, 200 μA) may have resulted not necessarily from activation of the STN but of surrounding neurons and/or fibers of passage. However, the activation of several fibers passing around STN are also considered as one of the mechanisms of STN-DBS and might also affect clinical symptoms in PD patients. We must also mention the length of stimulation in this study. If aim of this study was to examine the effect of STN on physiological activity, applying 10–15 s stimulation followed by a 1–2 min rest period and then followed by stimulation is preferable rather than applying long stimulation (30 min in this study), which might possibly cause neuronal fatigue or a lesion of stimulating sites. However, the aim of this study is to examine the effect of STN-DBS which is clinically used in PD patients by using PD model rat. In PD patients, impulse generator is implanted under the skin of the anterior chest, which deliver electrical stimulation to STN by connected wire continuously. Neuronal fatigue or a lesioning of STN is probably common in PD patients who underwent STN-DBS. In this study, we aimed to examine the effect of STN-DBS (which is clinically used in PD patients) on higher micturition center (mPFC in this study) by applying clinically used stimulation (long continuous stimulation and perhaps affect surrounding STN neurons and/or fibers of passage).

The present results show that STN-DBS increases bladder inter-contraction intervals by changing mPFC alpha power in normal and PD rats. The responses to STN-DBS in mPFC alpha power were different between normal and PD rats. Additionally, the levels of 5-HT and 5-HIAA decreased significantly in normal rats, while the levels of serotonin and dopamine and their metabolites decreased significantly in PD rats. STN-DBS might influence the activity of mPFC via thalamus and antidromic activation of hyper direct pathway, which might lead to prolongation of bladder inter-contraction interval through mPFC-PAG connection (Figure 5) (Fowler et al., 2008).

## Conclusion

STN-DBS can increase the inter-contraction interval of the bladder in normal and PD rats through changes in neural activity, as assessed by alpha power and catecholamine levels in mPFC. The effect of STN-DBS on catecholamine levels and the alpha power in mPFC differed between normal and PD rats.

## Data Availability Statement

The raw data supporting the conclusions of this article will be made available by the authors, without undue reservation.

## Ethics Statement

The animal study was reviewed and approved by The Guideline for the Care and Use of Laboratory Animals in Chiba University.

## Author Contributions

TY contributed conception and design of the study and wrote the first draft of the manuscript. RS and TU contributed conception and design of the study. SK supervised the first draft of the manuscript. All authors contributed to the article and approved the submitted version.

## Conflict of Interest

The authors declare that the research was conducted in the absence of any commercial or financial relationships that could be construed as a potential conflict of interest.
